# PA from a Recent H9N2 (G1-Like) Avian Influenza A Virus (AIV) Strain Carrying Lysine 367 Confers Altered Replication Efficiency and Pathogenicity to Contemporaneous H5N1 in Mammalian Systems

**DOI:** 10.3390/v12091046

**Published:** 2020-09-20

**Authors:** Ahmed Mostafa, Sara H. Mahmoud, Mahmoud Shehata, Christin Müller, Ahmed Kandeil, Rabeh El-Shesheny, Hanaa Z. Nooh, Ghazi Kayali, Mohamed A. Ali, Stephan Pleschka

**Affiliations:** 1 Center of Scientific Excellence for Influenza Viruses, National Research Centre (NRC), 12622 Giza, Egypt; sarahussein9@yahoo.com (S.H.M.); shehata_mmm@hotmail.com (M.S.); kandeil_a@hotmail.com (A.K.); ra_eny@yahoo.com (R.E.-S.); 2 Institute of Medical Virology, Justus Liebig University Giessen, Schubertstrasse 81, 35390 Giessen, Germany; christin.mueller@viro.med.uni-giessen.de; 3 St. Jude Children’s Research Hospital, 262 Danny Thomas Place, Memphis, TN 38105, USA; 4 Anatomy and Embryology Department, Faculty of Medicine, Jouf University, Sakaka 2014, Saudi Arabia; drhanaanooh@gmail.com; 5 Department of Epidemiology, Human Genetics, and Environmental Sciences, University of Texas, Houston, TX 77030, USA; ghazi@human-link.org; 6 Human Link, Baabda 1109, Lebanon

**Keywords:** avian influenza, H5N1, H9N2, R367K, reassortment, pathogenicity

## Abstract

Egypt is a hotspot for H5- and H9-subtype avian influenza A virus (AIV) infections and co-infections in poultry by both subtypes have been frequently reported. However, natural genetic reassortment of these subtypes has not been reported yet. Here, we evaluated the genetic compatibility and replication efficiency of reassortants between recent isolates of an Egyptian H5N1 and a H9N2 AIV (H5N1_EGY_ and H9N2_EGY_). All internal viral proteins-encoding segments of the contemporaneous G1-like H9N2_EGY_, expressed individually and in combination in the genetic background of H5N1_EGY_, were genetically compatible with the other H5N1_EGY_ segments. At 37 °C the replication efficiencies of H5N1_EGY_ reassortants expressing the H9N2_EGY_ polymerase subunits PB2 and PA (H5N1_PB2-H9N2EGY,_ H5N1_PA-H9N2EGY_) were higher than the wild-type H5N1_EGY_ in Madin-Darby canine kidney (MDCK-II) cells. This could not be correlated to viral polymerase activity as this was found to be improved for H5N1_PB2-H9N2EGY,_ but reduced for H5N1_PA-H9N2EGY_. At 33 °C and 39 °C, H5N1_PB2-H9N2EGY_ and H5N1_PA-H9N2EGY_ replicated to higher levels than the wild-type H5N1_EGY_ in human Calu-3 and A549 cell lines. Nevertheless, in BALB/c mice both reassortants caused reduced mortality compared to the wild-type H5N1_EGY_. Genetic analysis of the polymerase-encoding segments revealed that the PA_H9N2EGY_ and PB2_H9N2EGY_ encode for a distinct uncharacterized mammalian-like variation (367K) and a well-known mammalian signature (591K), respectively. Introducing the single substitution 367K into the PA of H5N1_EGY_ enabled the mutant virus H5N1_PA-R367K_ to replicate more efficiently at 37 °C in primary human bronchial epithelial (NHBE) cells and also in A549 and Calu-3 cells at 33 °C and 39 °C. Furthermore, H5N1_PA-R367K_ caused higher mortality in BALB/c mice. These findings demonstrate that H5N1 (Clade 2.2.1.2) reassortants carrying internal proteins-encoding segments of G1-like H9N2 viruses can emerge and may gain improved replication fitness. Thereby such H5N1/H9N2 reassortants could augment the zoonotic potential of H5N1 viruses, especially by acquiring unique mammalian-like aa signatures.

## 1. Introduction

Highly pathogenic avian influenza A viruses (HPAIVs) of the H5N1 subtype have crossed the animal/human barrier in 1997 causing lethal zoonotic infections in humans [1]. During the second wave of human HPAIV H5N1 infections—specifically in December 2005, HPAIV H5N1 (clade 2.2.1) have been reported in Egypt in migratory birds [2]. Subsequently in mid-February 2006, HPAIV H5N1 were transmitted to farmed and backyard poultry in Egypt [3,4] where they kept circulating. They were therefore officially declared “endemic” in the Egyptian poultry sector since 2008 [5]. In parallel, human infections with HPAIV H5N1 are annually reported since 2006, classifying Egypt as the country with the highest cumulative number of human cases in the world [1].

Concomitantly, low pathogenic avian influenza A virus (LPAIV) of the H9N2 subtype are circulating extensively worldwide in poultry for several decades, resulting in a high genetic diversity [1,6]. In 2010, LPAIV H9N2 of a G1-like lineage were detected in different avian species in Egypt [7,8,9]. Currently, the virus appears to be endemic in Egyptian poultry [6]. Since 2011, these viruses crossed the species barrier to humans due to their mammalian-like characteristics, causing mild to moderate human infections in different countries of the Middle East including Egypt [1,6]. The global concern about these H9N2 viruses is mainly associated with their ability to donate their genes to other avian influenza viruses (AIV) giving rise to high and low pathogenic AIVs that might thereby achieve the capability to efficiently cross the specie barrier and infect humans such as the LPAIV H7N9, reported in China since 2013 [1,10].

As both LPAIV H9N2 and HPAIV H5N1 subtypes are co-circulating in birds in several regions of the world co-infections with both subtypes have been frequently reported in individual avian hosts [11]. Consequently, this scenario increases the probability of genetic reassortment between both strains and could result in reassortants with enhanced zoonotic potential [10]. The limited detection of such reassortants in nature is probably due to insufficient surveillance, genetic incompatibility of possible H5N1/H9N2 segment combinations/constellations and/or negative selective pressure against such H5N1/H9N2 reassortants with new genetic constellations that render the virus replication incompetent, compared to the parental strains.

To better validate this potential threat, the present study set out to investigate the genetic compatibility and replication efficiency of reassortants, derived from recent avian influenza A/H5N1 and A/H9N2 viruses in vitro and pathogenicity in BALB/c mice. Moreover, a novel mammalian-like marker for avian influenza A viruses (IAVs) in the polymerase acidic (PA) protein was also described and characterized.

## 2. Materials and Methods

### 2.1. Cells, Viruses and Plasmids

Madin-Darby canine kidney (MDCK-II) cells, human lung adenocarcinoma epithelial cells (A549), human embryonic kidney cells (293T) and cultured human airway epithelial cells (Calu-3) were maintained in Dulbecco’s modified Eagle medium (DMEM) (Invitrogen, Carlsbad, CA, USA) supplemented with 100 I.U./mL penicillin, 100 μg/mL streptomycin (P/S) and 10% fetal bovine serum (FBS). All cell monolayers were incubated at 37 °C in a humidified CO_2_ incubator.

The highly pathogenic avian influenza virus A/chicken/Egypt/N12640A/2016(H5N1), designated hereafter as H5N1_EGY_, and the low pathogenic virus A/chicken/Egypt/S12568C/2016(H9N2), designated hereafter as H9N2_EGY_, were isolated from infected chicken in Egypt in 2016 by the Center of Scientific Excellence for Influenza Viruses (CSEIV), National Research Centre, Egypt. Both H5N1_EGY_ and H9N2_EGY_ were plaque-purified and propagated in the allantoic fluids of 10-day-old specific-pathogen-free (SPF) embryonated eggs. Inoculated eggs were incubated for 48 h at 37 °C and then chilled at 4 °C for 4 h before harvesting. Harvested stocks were titred by focus forming assay as previously described [12] and stored at −80 °C.

Plasmids expressing the eight viral segments of human influenza virus A/Anhui/1/2013(H7N9) designated H7N9Anhui [13], pMP-PB2-anhui, pMP-PB1-anhui, pMP-PA-anhui, pMP-HA-anhui, pMP-NP-anhui, pMP-NA-anhui, pMP-M-anhui and pMP-NS-anhui, were obtained from the plasmid stocks at the Institute of Medical Virology, Justus Liebig University Giessen, Germany.

Complete sets of pMP*ccd*B plasmids encoding the eight viral segments of H5N1_EGY_ and H9N2_EGY_ were constructed as previously described [14,15,16]. Briefly, the full length sequences of the 8 viral segments were amplified by RT-PCR using universal set of primers [14,16] and cloned into pMP*ccd*B as previously described [14].

### 2.2. Reverse Genetics (Rg) Systems for H5N1_EGY_ and H9N2_EGY_ and Generation of Reassortant, Mutant and Wild-Type Strains

**Figure 1:**
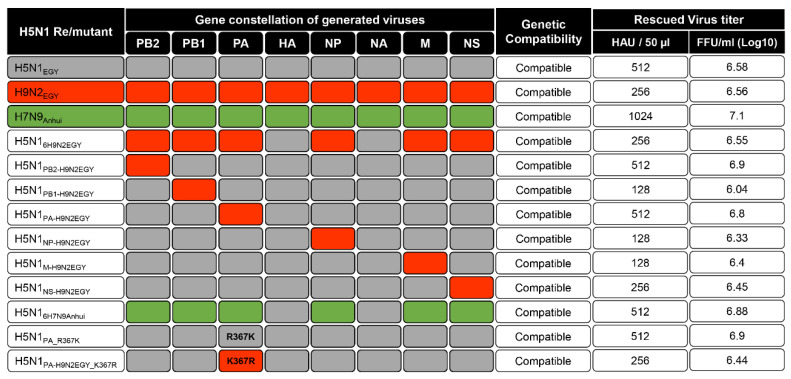
Genetic compatibility between Egyptian H5N1- and H9N2-viruses. The six internal-proteins encoding segments of LPAIV H9N2_EGY_, were placed individually and in combination, into the genetic background of HPAIV H5N1_EGY_. The gene segments from H5N1_EGY_ and H9N2_EGY_ are colored in grey and red, respectively. The six internal-proteins encoding segments from H7N9_Anhui_ (control) are colored in green. All genetic constellations used in this study were transfected to co-culture of 293T/MDCK-II cells and the rescued wild-type, reassortant and mutant viruses were propagated on embryonated SPF eggs. All genetic combinations were compatible showing variable hemagglutination unit (HAU) and focus forming unit (FFU) titers.

To generate wild-type strains and H5N1-subtype reassortants with specific genetic constellations (Figure 1), 8 μg of plasmid DNA (1 μg of each plasmid) encoding different combinations of the eight viral segments were transfected into a co-culture of 293T/MDCK-II cells (ratio 3:1) as previously described [14,17,18]. The transfection mixture was harvested 72 h post-transfection (p.t.). For LPAIV H9N2_EGY_, 1 µg/mL of L-(tosylamido-2-phenyl) ethyl chloromethyl ketone (TPCK)-treated trypsin was added to the transfection mixture 24 h p.t. and the transfection mixture was then harvested 48 h after addition of TPCK-treated trypsin. An aliquot of 100 µL of each supernatant was used to inoculate SPF embryonated eggs for another 48 h. The harvested viruses of compatible genetic constellations (Figure 1), were titrated using focus forming assay, titred, aliquoted and stored at −80 °C for further use.

Mutations of interest were introduced into the corresponding PA genes by using QuikChange site-directed mutagenesis (Agilent, Santa Clara, CA, USA) and specific mutagenesis primers (Table 1) according to manufacturer instructions. Sanger sequencing was performed to confirm the introduction of these mutants. Variant viruses H5N1_EGY_R367K_ and H5N1_PA-H9N2Egy_K367R_ carrying polymerase acidic (PA)-encoding segments with specific mutations were rescued individually by transfecting a co-culture of 293T/MDCK cells with eight pMP*ccd*B plasmids corresponding to the unchanged seven segments of the virus and the mutated one (7 WT viral segments + 1 mutant PA viral segment), using Trans-IT2020 (Mirus Bio, Madison, WI, USA) as described previously [14,19]. Recombinant viruses were rescued as described above and were subsequently sequenced to confirm the correct variant PB2 and PA segment in the genetic backbone of the other seven segments. Stocks of viruses harvested from infected MDCK-II cells were titrated by focus forming assay and kept at −80 °C until further use.

### 2.3. In Vitro Replication Efficiency of Reassortant and Wild-Type Strains

To determine the monocycle and multicycle replication efficiency of reassortant and wild-type strains, confluent monolayers of MDCK-II cells were infected in triplicate with reassortant and wild-type strains in PBS/BA (phosphate-buffered saline (PBS) containing 0.2% bovine albumin (BA), 1 mM MgCl_2_, 0.9 mM CaCl_2_, P/S) at multiplicity of infection (MOI) of 0.01 in a humidified CO_2_ incubator at 37 °C. The reassortant H5N1_6H7N9Anhui_, carrying the mammalian adapted H9N2-derived internal proteins-encoding segments (PB2, PB1, PA, NP, M, NS) of human influenza H7N9_Anhui_ virus and the HA and NA of H5N1_EGY_, was included as a positive control. After 1 h of virus-cell incubation at room temperature (RT), the inoculum was discarded and cell monolayers were washed twice with PBS^++^ (PBS containing 100 mg/l CaCl_2_ and 100 mg/l MgCl_2_) and replaced with infection medium DMEM/BA (DMEM, supplemented with 0.2% BA, 1% P/S and 1 μg/mL TPCK-treated trypsin). Supernatants were collected at 8 (monocycle) and 24 (multicycle) h post-infection (p.i.) and stored at − 80 °C.

### 2.4. Flow Cytometry Analysis of Viral Polymerase Activity

To analyze the effect of H9N2_EGY_ polymerase subunits on the overall H5N1_EGY_ viral polymerase expression activity, the pPOLI-GFP-RT plasmid was used as a reporter gene. The pPOLI-GFP-RT plasmid expresses a viral RNA (vRNA)-like POLI-transcript encoding the green fluorescent protein plasmid [20]. The viral ribonucloprotein complex (vRNP), composed of the different combinations of the co-expressed polymerase subunits and nucleoprotein, replicate and transcribe the (vRNA)-like POLI-transcript expressed by pPOLI-GFP-RT into GFP mRNA. The expressed GFP activity in the transfected cells is corresponding to the replication/transcription efficiency of the viral polymerase subunits. Briefly, 70–80% confluent monolayers of 293T cells were transfected with pPOLI-GFP-RT (2 µg) and four pMP*ccd*B expression plasmids encoding the PB1 (1 µg), PB2 (1 µg), PA (1 µg), and NP (2 µg) proteins from either H5N1_EGY_, H7N9_Anhui_ or H9N2_EGY_ and combinations thereof. Further, 293T cells, transfected only with pPOLI-GFP-RT (2 µg) were used as a control. The transfection mixtures were incubated at 37 °C and 5% CO_2_ for 8 h. Afterwards, the transfection mixtures were replaced with 2 mL Opti-MEM containing 0.2% BA and 1% P/S. GFP expression was monitored daily using fluorescent microscope (Carl Zeiss, Oberkochen, Germany). At 48 h p.t., the control and transfected cells were washed and harvested individually in PBS. A volume of 200 μL of each cell sample was mildly centrifuged and the supernatants were discarded. The cell pellets were re-suspended in 300 μL of FACS buffer (PBS with 0.5–1% BSA or 5–10% FBS, and 0.1% NaN3 at 1:10 ratio) and analyzed for percentage of GFP positive cells with fluorescein isothiocyanate (FITC)-channel using BD LSRFortessa cell analyzer (BD Biosciences, San Jose, CA, USA). The control 293T cells were used as negative control to eliminate any auto-fluorescence.

### 2.5. Temperature-Dependent Replication Kinetics of Reassortant and Wild-Type Strains

In order to determine the effect of temperature on the replication ability of the reassortant viruses, low MOI growth curves were carried out in triplicate by infecting A549 and Calu-3 cell cultures in 6-well plates at an MOI of 0.001. The reassortant H5N1_6H7N9Anhui_ was included as a positive control. In parallel, cryopreserved normal human bronchial epithelial (NHBE) cells were seeded and differentiated as described previously [21]. Briefly, undifferentiated cells were seeded on transwell plates (Corning, New York, NY USA) and grown in a mixture of DMEM (Invitrogen) and Bronchial Epithelial Cell Growth Basal Medium (BEGM) (Lonza, Köln, Germany) supplemented with retinoic acid (75 nM). Confluent cell layers were further differentiated under air-liquid interface conditions for at least 4 additional weeks until they resembled a pseudostratified human airway epithelia. Cells were then infected with an MOI of 1. The infected cells were incubated with virus diluted in PBS^++^ for 1 h at RT. The infection inocula were then aspirated, the cells washed twice with PBS^++^ and supplemented with 2 mL of infection (DMEM/BA) medium for 36 h at 37 °C (NHBE) 33 °C and 39 °C (A549 and Calu-3). Supernatants were collected at 6, 12, 24 and 36 h p.i. and stored for further use at −80 °C.

### 2.6. Pathogenicity of Reassortant Strains versus Wild-Type H5N1_EGY_ in BALB/c Mice

To determine body weight and survival rate in vivo, female BALB/c mice (6–8 week-old) were divided into four groups (10 mice/group). Three groups of mice were anesthetized with isoflurane and intranasally inoculated with a challenge dose (10^5^ FFU in 100 µL PBS) of each virus “H5N1_EGY,_ H9N2_EGY_, H5N1_PB2-H9N2EGY_, or H5N1_PA-H9N2EGY_” and an uninfected control group was anesthetized and intranasally inoculated with 100 µL of 1X PBS. Infected and control mice were then monitored for 14 days post-infection (dpi) for body weight loss and survival rate. Mortality was recorded either as an actual death or loss of ≥25% of initial body weight, which is the threshold at which the animals were humanely euthanized.

### 2.7. Luciferase Reporter Assay

The Luciferase reporter gene assay was performed as previously described [13] with minor modifications. Briefly, 293T (2 × 10^6^ cells/well) were seeded in 6-well plates and co-transfected with 1 µg of each pMP*ccd*B expression plasmid encoding the individual viral ribonucleoprotein complex (vRNP; PB2, PA, PB1 and NP) components of H5N1_EGY,_ or H9N2_EGY_, or H7N9_Anhui_, or vRNP components of H5N1_EGY_ expressing individually the mutated PA_R367K of H5N1_EGY_ or PA_K367R of H9N2_EGY_. The four expression plasmids were mixed with 40 ng pRL-SV40 (Renilla luciferase expression plasmid for normalization), and 200 ng of a firefly luciferase reporter plasmid pHW72-Luc expressing negative-sense firefly luciferase flanked by noncoding sequences of NS segment under the control of a species-specific polymerase I promoter. Transfection was performed using Trans-IT2020 as previously described [14] for 8 h. After 24 h of transfection, the cells were harvested, washed one time with 1x PBS, and lysed with 200 μL of “1x passive lysis buffer” (Promega, Madison, WI, USA). The Firefly/Renilla luciferases were quantified using a Dual-Luciferase Reporter Assay System (Promega) and measured using a Spark 10M multimode microplate reader (TECAN). Relative luminometer units (RLU), normalized to Renilla luciferase, refer to fold induction of polymerase activity.

### 2.8. Pathogenicity of Mutated Strains versus Wild-Type H5N1_EGY_ in BALB/c Mice

To investigate the role of the 367K in the pathogenesis of H5N1_EGY_ infection in vivo, female BALB/c mice (6–8 week-old) were divided into six groups (10 mice/group). Five groups of mice were anesthetized with isoflurane and intranasally inoculated with a challenge dose (10^3^ FFU in 30 µL PBS) of each mutant versus the corresponding reassortant and wild-type H5N1_EGY_ and H9N2_EGY_ viruses. An uninfected control group was anesthetized and intranasally inoculated with 30 µL of 1X PBS. Infected and control mice were then monitored for 14 dpi for body weight loss and survival rate. Mortality was recorded either as an actual death or loss of ≥25% of initial body weight, which is the threshold at which animals were humanely euthanized.

### 2.9. Ethical Statement and Biosafety

All animal experiments and procedures were conducted in accordance with the guidelines and regulations of approved by the Medical Research Ethics Committee (MREC) of the National Research Centre (NRC), Egypt (permission code: 16–247; permission date: 1 August, 2016). All experiments using infectious virus were performed in accordance with the Egyptian and German biosafety regulations pertaining to the propagation of influenza viruses. All experiments involving IAVs were performed using Biosafety Level 3 containment laboratories approved for such use by the local authorities (RP, Giessen, Germany).

### 2.10. Statistical Analysis

Statistical analysis and graphical data presentations were performed using GraphPad Prism 5.0 software (GraphPad Software, San Diego, CA, USA). Significances were determined either with repeated-measures ANOVA with Bonferroni’s post-hoc test, one-way analysis of variance ANOVA followed by Dunnett’s multiple comparison post hoc Test, one-way ANOVA followed by Tukey’s post-hoc test or a two-tailed unpaired t-test with Welch’s correction as indicated in each relevant figure legend. All data are presented as mean ± SD.

## 3. Results

### 3.1. Gene segments of H9N2_EGY_ Show High Genetic Compatibility in the Genetic Background of H5N1_EGY_

HPAIV H5N1_EGY_ and LPAIV H9N2_EGY_ were isolated from infected chicken in Egypt in 2016. Both strains have no history of previous adaptation to mammalian cell culture systems; therefore, they were selected to study the effect of H9N2_EGY_ gene segments in genetic background of H5N1_EGY_ on the replication ability, polymerase activity in vitro and virulence in mammalian systems in vivo.

To investigate these aspects we successfully established complete reverse genetic (Rg) systems for both wild-type strains and specific H5N1_EGY_ reassortants, expressing the internal proteins-encoding segments of H9N2_EGY_ individually or in combination (Figure 1), were generated. Additionally, a recombinant H5N1 reassortant expressing mammalian-adapted internal protein-encoding segments of the human influenza virus isolate A/Anhui/1/2013 (H7N9_Anhui_) and the HA and NA of H5N1_EGY_ was generated as a control. This control represents a reassortant H5N1 virus carrying internal protein-encoding segments derived from a BJ16-like H9N2-subtype AIV [22] as most of the human H9N2 isolates were genetically classified either as G1- or as BJ16-like H9N2 [6].

Interestingly, all reassortants were rescued indicating that all genetic combinations were compatible in the genetic background of H5N1_EGY_ indicating the high genetic compatibility between H9N2_EGY_ and H5N1_EGY_.

### 3.2. Improved Replication Efficiency of H5N1_EGY_ Reassortants Expressing PB2 and PA of H9N2_EGY_ in MDCK-II Cells

**Figure 2:**
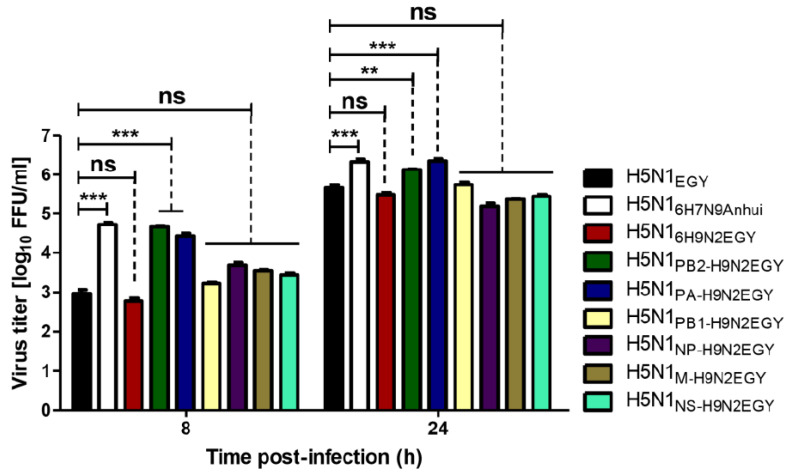
Replication efficiency of the H5N1_EGY_ reassortants. Mammalian Madin-Darby canine kidney (MDCK-II) cells were infected (in triplicate) with H5N1_EGY_ reassortants expressing internal proteins-encoding H9N2_EGY_ genes or with the wild-type H5N1_EGY_ or the control H5N1_6H7N9Anhui_ virus at MOI of 0.01, cultured at 37 °C for single replication cycle (8 h) and multiple replication cycles (24 h) p.i. Subsequently the virus titers were determined. Error bars reflect standard deviation (SD) of three independent experiments. Statistical analysis was performed using repeated measures ANOVA, followed by Bonferroni post hoc test. The significant differences are indicated (** = *p* < 0.01, *** = *p* < 0.001 and non-significant = ns).

The replication efficiencies of H5N1_EGY_ reassortants were characterized by infection of mammalian MCDK-II cells at a low multiplicity of infection (MOI) = 0.01 at 37 °C for 8 h (single replication cycle) and 24 h (multi-replication cycles). Out of the 7 reassortants, only two H5N1 reassortants, which express the PB2 and PA polymerase subunits of H9N2_EGY_ (H5N1_PB2-H9N2EGY_ and H5N1_PA-H9N2EGY_) replicated significantly higher than the parent H5N1_EGY_ strain (Figure 2). The replication efficiencies of the other H5N1_EGY_ reassortants carrying gene segments of H9N2_EGY_ were comparable or lower than those of the H5N1_EGY_ and the control H5N1_6H7N9Anhui_.

### 3.3. Impact of the Polymerase Subunits of H9N2_EGY_ on the Polymerase Activity of Reassortant H5N1_EGY_

**Figure 3:**
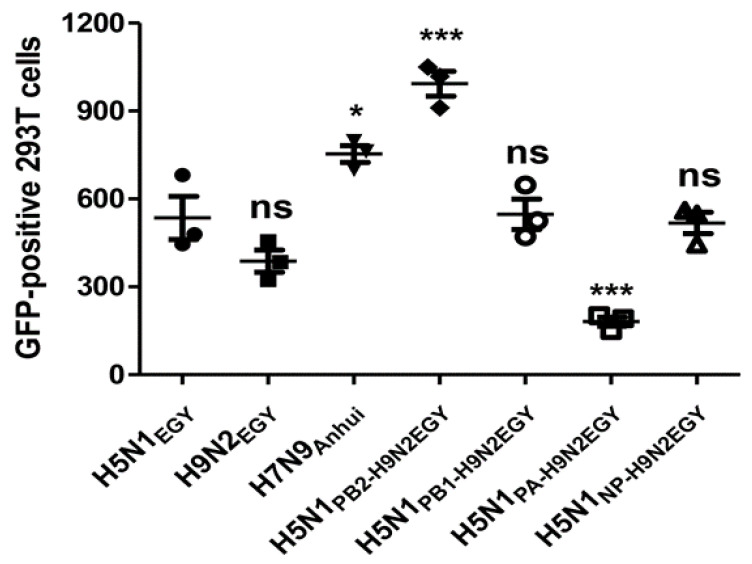
In vitro polymerase activity in human 293T cells. 293T cells were transfected with plasmids expressing the three subunits (PB2, PB1, and PA) of the viral RNA-dependent RNA polymerase (RdRp) and the viral nucleoprotein (NP) of H5N1_EGY_, H9N2_EGY,_ H7N9_Anhui_ or combinations of H5N1_EGY_ RdRp subunits with single RdRp subunits of H9N2_EGY_. Along with the three RdRp subunits and NP expressing plasmids, a vector expressing a vRNA-like Pol-1 transcript encoding the reporter GFP gene was co-transfected. At 48 h p.t., the control and the transfected cells were analyzed for percentage of GFP positive cells. The significance was tested using one-way ANOVA, followed by Dunnett’s multiple comparison post hoc test and the significant differences are indicated (* = *p* < 0.05, *** = *p* < 0.001 and non-significant = ns).

To investigate whether the enhanced in vitro replication efficiency for H5N1_PB2-H9N2EGY_ and H5N1_PA-H9N2EGY_ was accompanied with an improved polymerase activity of the reassortants, a mini-genome assay using combinations of PB2, PB1, PA, and NP of the parent strains, H5N1_EGY_ and H9N2_EGY_, together with a vRNA-like transcript expressing the GFP-reporter gene followed by flow cytometry analysis was applied. Interestingly, the polymerase comprising PB1, PA and NP of H5N1_EGY_ and the PB2 of H9N2_EGY_ showed a significant activity increase compared to the activities of either the parental vRNP-H5N1_EGY_ or vRNP-H9N2_EGY_ polymerases (Figure 3). Yet, the polymerase activity constituted by the PB2, PB1 and NP of H5N1_EGY_ together with the PA of H9N2_EGY_, was significantly lower than the polymerase activities of the parental polymerases of either H5N1_EGY_ or vRNP-H9N2_EGY_. This indicates that the PA protein of H9N2_EGY_ improves H5N1_EGY_ fitness by other mechanisms, rather than improving the polymerase activity of the H5N1_PA-H9N2EGY_ reassortant strain.

### 3.4. Replication Efficiency of Reassortant and Wild-Type H5N1_EGY_ in Human Lung Cells Increases at Elevated Temperature Resembling Lung Ambient Temperature in Humans

In an attempt to better estimate the outcome of human infections with reassortants between H5N1_EGY_ and H9N2_EGY_, we set out to examine the influence of site-specific mammalian body temperatures on viral replication efficiency. Therefore, we investigated whether different temperatures resembling either the initial site of IAV replication in the upper respiratory tract (33 °C) or the later replication site of the lower respiratory tract (39 °C) in case of viral pneumonia [23], would affect the replication efficiency of the H5N1_PB2-H9N2EGY_ and H5N1_PA-H9N2EGY_ versus wild-type H5N1_EGY_ and the control H5N1_6H7N9Anhui_. To this point, mammalian cell lines Calu-3 as an in vitro model for bronchial epithelial cells of the upper respiratory tract (URT) [24] and A549 as a type-II alveolar epithelial cell model of the lower respiratory tract (LRT) [25] were infected in triplicates with the indicated viruses at low MOI of 0.001.

**Figure 4:**
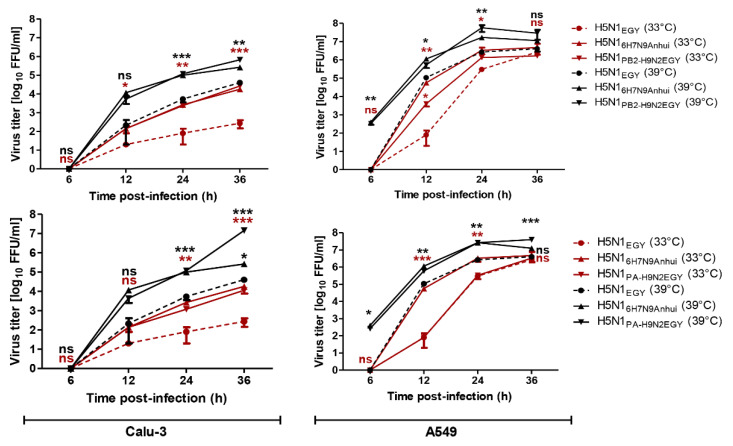
Replication kinetics of H5N1_EGY_ reassortants in mammalian cell culture models. Calu-3 and A549 cells were infected (in triplicates) with the H5N1_EGY_ reassortants or wild-type H5N1_EGY_ and control H5N1_6H7N9Anhui_ at multiplicities of infection (MOIs) of 0.001 and incubated at 33 °C or 39 °C. At 6, 12, 24, 36 h p.i., the cell culture supernatants were collected and the virus titre was determined. Statistical analysis was performed using two-way ANOVA, followed by Bonferroni post hoc test. The significant differences are indicated (* = *p* < 0.05, ** = *p* < 0.01, *** = *p* < 0.001 and non-significant = ns).

The obtained results (Figure 4) demonstrated that in Calu-3 cells both reassortants H5N1_PB2-H9N2EGY_ and H5N1_PA-H9N2EGY_ replicate to higher levels than the parental H5N1_EGY_, but comparable to the control H5N1_6H7N9Anhui_ at both temperature settings. In A549 cells, the H5N1_PB2-H9N2EGY_ replicated significantly higher than the wild-type H5N1_EGY_, but comparable to control H5N1_6H7N9Anhui_ at 33 °C and 39 °C. However, the replication of H5N1_PA-H9N2EGY_ in A549 cells was only higher than the wild-type H5N1_EGY_ at 39 °C. These data demonstrate the ability of both reassortant viruses to replicate efficiently at different temperature conditions, mimicking the temperatures of the different lung compartments during AIV invasion at the initial site of infection and subsequently during alveolar infection.

### 3.5. Reassortants H5N1_PB2-H9N2EGY_ and H5N1_PA-H9N2EGY_ Showed Similar to Lower Virulence When Compared to the Wild-Type H5N1_EGY_ in Mice

**Figure 5:**
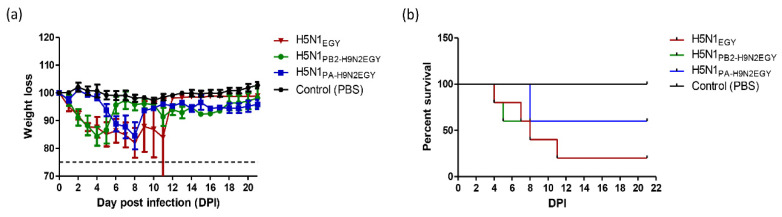
Pathogenicity of H5N1_EGY_ and reassortants H5N1_PB2-H9N2EGY_ and H5N1_PA-H9N2EGY_ in female *BALB*/*c* mice. Mice were infected with 10^5^ PFU of each virus in 100 μL phosphate-buffered saline (PBS). The morbidity rate and the mortality rate as demonstrated by weight loss of body (**a**) and the survival rate (**b**), respectively, were monitored for 14 dpi. Mice judged moribund (body weight loss >25%) were euthanized.

To analyze the characteristics of H5N1_PB2-H9N2EGY_ and H5N1_PA-H9N2EGY_ in vivo we inoculated female BALB/c mice with the reassortant viruses and the wild-type H5N1_EGY_. After inoculation (10^5^ PFU/100 µL) with H5N1_PB2-H9N2EGY_ and H5N1_EGY_ all mice showed comparable reduction in body weight at 1 dpi compared to the relative body weight immediately before infection and compared to the PBS control group (Figure 5a). Unlike H5N1_EGY_-infected mice_,_ which started to regain body weight at 11 dpi (2 mice only), the body weight of H5N1_PB2-H9N2EGY_ inoculated mice started to increase faster at 5 dpi to the end of the experiment. However, the H5N1_PA-H9N2EGY_ inoculated mice exhibited transient reduction in body weight at 5 dpi and the body weight started to increase at 9 dpi to the end of the experiment, although at significantly lower levels than the control group (Figure 5a). Interestingly, the survival rates between the reassortants and the wild-type differed strongly (Figure 5b). Here, 60% of the mice survived the infection with the reassortants, while only 20% of the mice survived the infection with the wild-type virus.

These data suggest that the reduced pathogenicity of the reassortant H5N1_PB2-H9N2EGY_ and H5N1_PA-H9N2EGY_ viruses may be attributed to specific genetic difference between the PA and PB2 segments of H9N2_EGY_ and H5N1_EGY_.

### 3.6. The Genetic Analysis of the Polymerase-Encoding Segments from H9N2_EGY_ Revealed that the PA_H9N2EGY_ and PB2_H9N2EGY_ Encode for Distinct Mammalian-Like Variations

To investigate the molecular basis behind the enhanced replication efficiency of H5N1_PB2-H9N2EGY_ and H5N1_PA-H9N2EGY_ in vitro, we compared the genetic differences between the PB2 and PA segments from H5N1_EGY_ with their corresponding segments in H9N2_EGY_. Genetic analysis revealed that the PB2 and PA of H5N1_EGY_ and H9N2_EGY_, namely PB2_H9N2EGY_ and PA_H9N2EGY_, shared 86% and 89.9% nucleotides and 96.4% and 95.4% amino acids (aa) identity, respectively. The PB2 and PA of H5N1_EGY_ and H9N2_EGY_ differ in 26 and 33 aa, respectively (Table 2).

**Figure 6:**
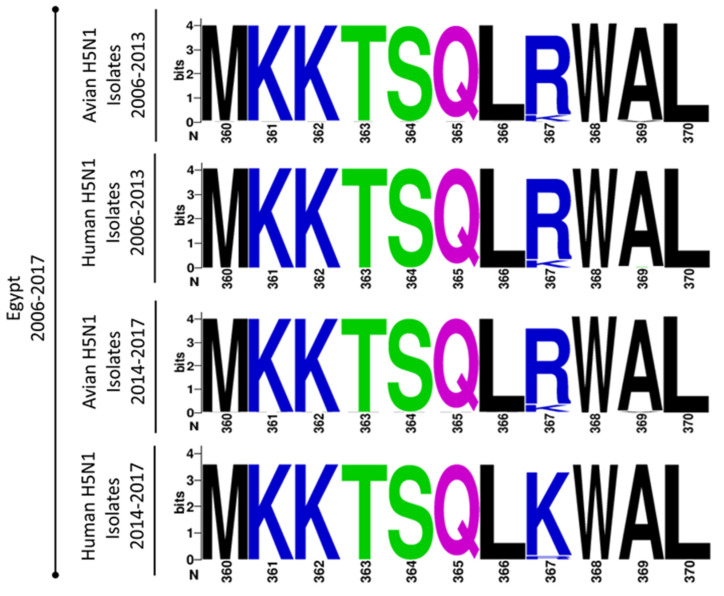
Prevalence of lysine and argenine at amino acid (aa) residue 367 among Egyptian human and avian H5N1 isolates (2006 to 2017). The graphic was created via Web-based WebLogo application (http://weblogo.threeplusone.com/create.cgi) [29]. The aa color is given according to their chemical properties. Polar aa “T and S”: green; neutral aa “Q”: purple; basic aa “K and R”: blue; hydrophobic aa “M, L, W, A and L”: black.

Except for the well-known mammalian signature 591K [26,27,28], and the yet un-described 367K (Figure 6) in the PB2_H9N2EGY_ and PA_H9N2EGY_, respectively, all other variations in the PB2_H9N2EGY_ and PA_H9N2EGY_ were predominant in the corresponding polymerase protein-encoding segments of circulating H9N2 and H5N1 AIV isolates, but were less frequently observed in human isolates of the same subtypes. Unlike Q591K [28], the impact of the R367K substitution in the PA on the replication efficiency, polymerase activity and virulence of H5N1 strains has not been characterized yet.

### 3.7. The H5N1_EGY_ Mutant Expressing PA_R367K_ Replicates Efficiently in Primary Human Bronchial Epithelial Cells and Continuous Human Cell Culture Models in a Temperature-Dependent Manner

**Figure 7:**
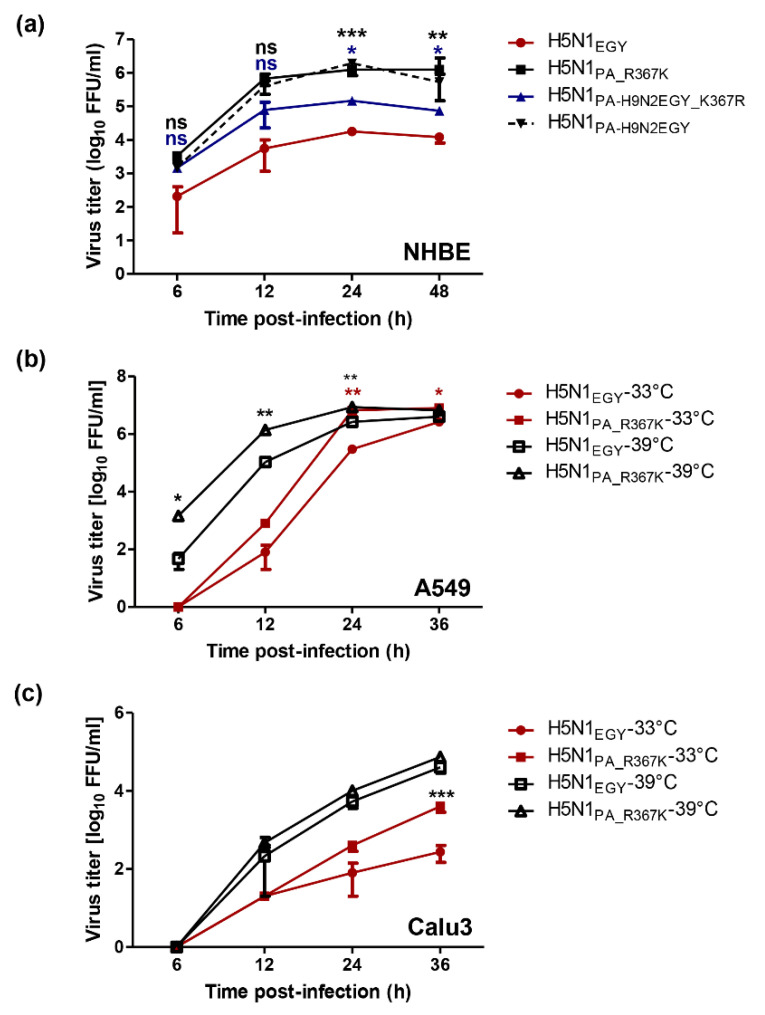
Impact of PA-367K on the replication efficiency of H5N1_EGY_ in mammalian cell culture models. (**a**) mammalian differentiated primary bronchial epithelial cells (NHBE) cells were infected (in triplicates) with wild-type H5N1_EGY_, reassortant H5N1_PA-H9N2EGY_, as well as mutant H5N1_PA_R367K_ and H5N1_PA-H9N2EGY_K367R_ at MOI of 1, cultured at 37 °C for 6–36 h p.i. (**b**) A549 and (**c**) Calu-3 were infected with wild-type H5N1_EGY_ and mutant H5N1_PA_R367K_ (MOI = 0.01) and cultured at 33 °C and 39 °C for 6–36 h p.i. Subsequently the virus titers were determined. Error bars reflect standard deviation (SD) of three independent experiments. Statistical analysis was performed using repeated measures ANOVA, followed by Bonferroni post hoc test. The significant differences are indicated (* = *p* < 0.05, ** = *p* < 0.01, *** = *p* < 0.001 and non-significant = ns).

To better understand the impact of lysine at position 367 in the PA of H9N2_EGY_, primary human bronchial epithelial (NHBE) cells were infected (MOI = 1) with wild-type H5N1_EGY_, H5N1_PA_R367K_ and H5N1_PA-H9N2EGY_K367R_ mutants and reassortant H5N1_PA-H9N2EGY_. At 37 °C, the reassortant H5N1_PA-H9N2EGY_ and the mutant H5N1_PA_R367K_ showed significant higher virus replication than the wild-type virus at 12–48 h p.t. (Figure 7a). The reassortant H5N1_PA-H9N2EGY_K367R_ demonstrated lower replication efficiency than the H5N1_PA-H9N2EGY_ and H5N1_PA_R367K_ viruses, but still higher than the H5N1_EGY_ (Figure 7a).

Based on the fact that the initial infection and onward transmission of an influenza virus depends on efficient virus replication in the human respiratory tract, we tested the replication of the mutant H5N1_PA_R367K_ versus the wild-type H5N1_EGY_ in A549 (Figure 7b) and Calu-3 (Figure 7c) at two different temperature settings, 33 °C and 39 °C, representing the initial site of infection and the subsequent alveolar infection, respectively. Interestingly, H5N1_PA_R367K_ showed significant higher replication than the wild-type H5N1_EGY_ in A549 cells at 12–24 h p.i. at 39 °C and 24–36 h p.i. at 33 °C (Figure 7b). In Calu-3 cells, comparable replication kinetics were observed for H5N1_PA_R367K_ and H5N1_EGY_ at 39 °C. However, H5N1_PA_R367K_ showed significant higher replication than the wild-type at 36 h p.i. at 33 °C (Figure 7c). This indicates a possible improvement of the viral fitness of H5N1_PA_R367K_ at the initial site of infection.

### 3.8. Replication Efficiency of H5N1_PA_R367K_ Is Not Associated with an Enhanced Polymerase Activity

**Figure 8:**
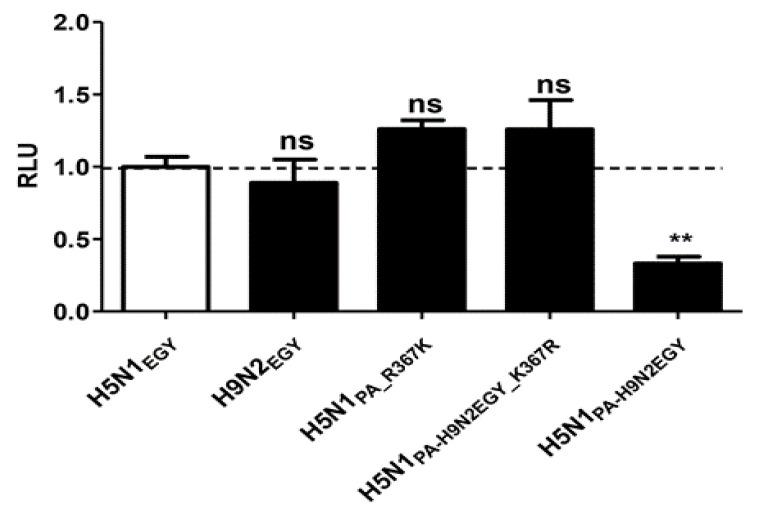
PA-367K does not significantly alter the in vitro polymerase activity of H5N1_EGY_ in human 293T cells. The 293T cells were transfected with plasmids expressing the three subunits (PB2, PB1, PA) of the viral RNA-dependent RNA polymerase (RdRp) and the viral nucleoprotein (NP) of H5N1_EGY_ or H9N2_EGY_ (controls) or combinations of H5N1_EGY_ PB2, PB1 and NP with mutated PA of H5N1_EGY_ (PA_R367K) or of H9N2_EGY_ (PA-H9N2EGY_K367R), as well as wild-type PA of H9N2_EGY_ (PA-H9N2EGY). Along with the three RdRp subunits and NP expressing plasmids, a Renilla luciferase expression plasmid (transfection control) and a vector expressing a vRNA-like Pol-1 transcript encoding the firefly luciferase was co-transfected. At 48 h p.t., the control and transfected cells were analyzed for Renilla/luciferase expression levels. The significance was tested using one-way analysis of variance ANOVA, followed by Dunnett’s multiple comparison post hoc test and the significant differences are indicated (** = *p* < 0.01 and non-significant = ns).

To investigate whether the improved replication efficiency of H5N1_PA_R367K_ in mammalian cell culture models is linked to an improved polymerase activity, we further examined and compared the effect of the PA mutations in a mini-genome reporter assay using plasmids expressing the three subunits (PB2, PB1, PA) of the viral RNA-dependent RNA polymerase (RdRp) and the viral nucleoprotein (NP) of H5N1_EGY_ or H9N2_EGY_ (controls) or combinations of H5N1_EGY_ PB2, PB1 and NP with mutated PA of H5N1_EGY_ (PA_R367K) or of H9N2_EGY_ (PA-H9N2_EGY__K367R), as well as wild-type PA of H9N2_EGY_ (PA-H9N2_EGY_). Along with the three RdRp subunits and NP expressing plasmids, a Renilla luciferase expression plasmid (transfection control) and a vector expressing a vRNA-like Pol-1 transcript encoding the firefly luciferase was co-transfected (Figure 8). The results show that both the H5N1_EGY_ PA_R367K and the H5N1_EGY_ PA-H9N2_EGY__K367R slightly improved the polymerase activity of the Renilla luciferase expression by the H5N1_EGY_ vRNP with PA alterations, but not significantly when compared to the wild type H5N1_EGY_ vRNP. The fact that the H9N2_EGY_ PA-H9N2_EGY_ reduced the H5N1_EGY_ vRNP Renilla luciferase expression activity correlates well with the result of the mini-genome analysis with the GFP-expressing reporter gene (Figure 3). In the light of these data, we conclude that the improved replication efficiency of H5N1_PA_R367K_ is not directly related to an enhanced polymerase activity.

### 3.9. The PA_R367K Contributes to the Pathogenicity of H5N1_EGY_ in Mice

**Figure 9:**
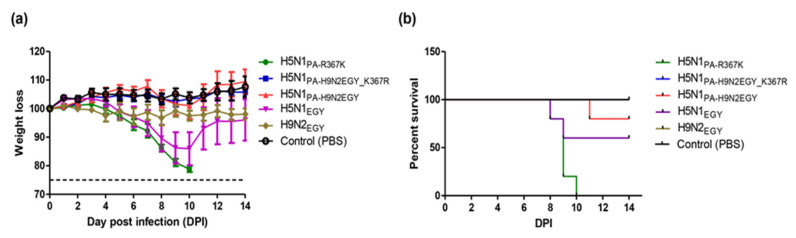
Pathogenicity of wild-type H5N1_EGY,_ H9N2_EGY_, reassortants H5N1_PB2-H9N2EGY_ and H5N1_PA-H9N2EGY_, mutants H5N1_PA_R367K_ and H5N1_PA-H9N2EGY_K367R_ in female BALB/c mice. Mice were infected with 10^3^ PFU of each virus in 30 μL PBS. (**a**) The body-weight reduction rate and the mortality rate as demonstrated by weight loss (**a**) and the survival rate (**b**), respectively, were monitored for 14 dpi. Mice judged moribund (body weight loss > 25%) were euthanized.

To examine the effect of R367K mutation in the H9N2_EGY_ to the pathogenicity in mice, we intranasally infected 6 to 8-week-old healthy female BALB/c mice (10 mice in each group) with a lower infectious dose (10^3^ PFU) in 30 µL PBS of the recombinant wild-type strains (H5N1_EGY_ and H9N2_EGY_), the reassortant (H5N1_PA-H9N2EGY_), and the H5N1 expressing PA_H5N1EGY_R367K_ (H5N1_PA_R367K_) or the mutated PA_H9N2EGY_K367R_ (H5N1_PA-H9N2EGY_K367R_). Mice inoculated with these viruses were monitored for 14 days for weight loss and mortality (Figure 9a). The mice infected with H9N2_EGY_, H5N1_PA-H9N2EGY_ and H5N1_PA-H9N2EGY_K367R_ virus showed less than 5% of weight loss in the next 14 days p.i. The mice demonstrated around 10–20% of weight loss after infection with the wild-type H5N1_EGY_ and the corresponding H5N1_PA_R367K_ mutant. Even though the pattern of body weight reduction was comparable between the H5N1_EGY_ and H5N1_PA_R367K_, the H5N1_EGY_-infected mice started to gain weight at day 9 p.i.

In contrast the survival rates of the mice infected with the different viruses varied strongly (Figure 9b). Here it showed that while all mice infected with H9N2_EGY_ and H5N1_PA-H9N2EGY_K367R_ survived, only 80% of H5N1_PA-H9N2EGY_-infected mice and 60% of H5N1_EGY_-infected mice survived. Most strikingly, all mice infected with H5N1_PA_R367K_ died without exceeding the 25% body weight reduction limit. These data suggest that the R367K substitution alone in the PA of H5N1_EGY_ could contribute to the enhanced mortality of H5N1_PA_R367K_ in mice.

## 4. Discussion

The genome of influenza viruses (IVs) shows a high genetic plasticity based on nucleotide exchange due to the error prone polymerase, as well as gene segment reassortment between different viruses upon co-infections with different virus strains [1]. This allows these viruses to escape selective pressures, such as antibodies, and to quickly adapt to new host species. For AIV these genome modifications can lead to a dynamic interspecies transmission resulting in the continuous emergence of unexpected AIV variants with a varying potential for a bird-to-human transmission [30]. Historically, influenza A virus (IAV) reassortants represent potential threats to the public health in the form of global pandemic influenza outbreaks [1,31]. Along this line, IAV segments encoding the viral internal proteins of H9N2-subtype LPAIV were recently found to augment the zoonotic potential of different AIV subtypes [1,10]. By successful employment of reverse genetics systems, we were able to demonstrate in this study that the gene segments derived from a contemporary G1-like H9N2-subtype AIV, isolated from chicken in Egypt in 2016, show a high genetic compatibility in the genetic background of a H5N1-subtype HPAIV, also isolated from chicken in Egypt in 2016. All seven possible H5N1 reassortants carrying a single H9N2-subtype internal gene segment individually (H5N1_PB2-H9N2EGY_, H5N1_PB1-H9N2EGY_, H5N1_PA-H9N2EGY_, H5N1_NP-H9N2EGY_, H5N1_M-H9N2EGY_ and H5N1_NS-H9N2EGY_) or in combination (H5N1_6H9N2EGY_) were compatible to the remaining H5N1_EGY_ segments and could be rescued. These results are in agreement with a recent study showing remarkably high genetic compatibility between a HPAIV H5N1-subtype (clade 2.2.1.2) and a LPAIV H9N2-subtype (G1-like) strain, both isolated in 2013 [32]. Nevertheless, another study, analyzing natural reassortment between A/chicken/Egypt/AR236/2015 (H5N1) and A/chicken/Egypt/AR755/2013 (H9N2) in co-infected embryonated chicken eggs reported the recovery of only five H5N1-subtype reassortants in a total of 100 plaque-picked viruses [33]. Yet, the authors did not exclude the possibility that more reassortants with different genetic constellations might have been present in the allantoic fluid of the mixing vessel [33]. Importantly, our study provides an additional evidence that, similar to H7N9-, H10N8- and H5N6-subtype AIVs [1,10], the H9N2-subtype viruses are able, to donate their internal-proteins encoding segments via a unidirectional genetic transfer to H5N1-subtype strains [33]. In complement to this study, we previously tested the genetic compatibility of viral segments derived from HPAI A/duck/Egypt/Q4596D/2012 (H5N1) (H5N1/Q4596D) in the genetic backbone of LPAI A/chicken/Egypt/S4456B/2011 (H9N2) (H9N2/S4456B) using Rg approach of influenza viruses [34]. Except for NP segment from H5N1/Q4596D, all internal protein-encoding segments were compatible with the complementary seven segments from H9N2/S4456B and viruses could be rescued [34]. Although the compatibility results is very likely a strain specific characteristic and cannot be expanded to the subtype as a general rule, the data provided in our work further indicate a high genetic compatibility between both investigated strains and support the assumption that H5N1/H9N2 reassortants have no genetic restrictions to emerge naturally in the populations.

In MDCK–II cells, only H5N1_PB2-H9N2EGY_ and H5N1_PA-H9N2EGY_ replicated significantly higher than the parental H5N1_EGY_, but comparable to the replication efficiency of H5N1_6H7N9Anhui_. The reassortant H5N1_6H7N9Anhui_ virus comprises the internal protein-encoding segments of the human AIV-isolate A/Anhui/1/2013(H7N9) strain (H7N9_Anhui_). Genetic analysis of the human 2013 H7N9 strains in China indicated that the internal-protein encoding segments were likely derived from a BJ16-like H9N2-subtype AIV [22]. Notably, most of the human H9N2 isolates were genetically classified as G1- or BJ16-like H9N2 [6]. This might explain the ability of the PB2 or PA segment from G1-like H9N2_EGY_ to improve the replication fitness of H5N1_EGY_ to similar levels as the H5N1_EGY_ expressing the internal-protein encoding segments of the human adapted BJ16-like H9N2 of H7N9_Anhui_.

Our data are partially consistent with those of Arai et al. showing that the replication of a reassortant H5N1-subtype HPAIV containing the NS and/or the PB2 or PA gene segment of a H9N2-subtype LPAIV tended to be higher in mammalian cells at 37 °C and 33 °C than that of the wild-type H5N1 strain [32]. Contrary to Arai et al., we showed that the H5N1_EGY_ reassortant expressing the NS proteins of H9N2_EGY_ propagated to comparable levels like the wild-type H5N1_EGY_ at least at 37 °C. By comparing the NS1 and NS2 of 2016 H9N2_EGY_ and A/chicken/Egypt/CL42/2013 (CL42) (H9N2) used by Arai et al., we found that they share 91.7% and 95% aa similarly, respectively. The NS1 proteins of our H9N2_EGY_ and CL42 strains belong to allele “A” and share the same pattern of mammalian-like markers including P42S and mammalian like PDZ motifs “ESKV for H5N1_EGY_ and KSEV for CL42”. Similar to our finding, Arai and his colleagues showed that the 6 internal-protein encoding segments or the NS segment alone of the H9N2-subtype LPAIV could also enhance the replication of the corresponding H5N1 reassortants to higher levels than the wild-type H5N1 strain, indicating that the in vitro replication was related to the internal gene cassette rather than the surface proteins-encoding segments [32]. Contrary to our data and the data shown by Arai et al., the natural reassortments studied by Naguib et al. have shown that the replication efficiency of natural H5N1 reassortants expressing the PB2 or the six internal-protein encoding segments of H9N2 (clone C46) were either retarded or unaltered, compared to the wild-type H5N1 strain [33]. This might indicate that only specific aa variations in the studied H5N1 and H9N2 strains in each study, collected on 2013, 2015 and 2016, might affect the impact of the reassortment on the replication efficiency of the corresponding H5N1-subtype reassortants.

To study the mechanism(s) underlying the replication differences determined for the two polymerase reassortants (PB2 and PA), the expression activity of different combinations of polymerase subunits and nucleoprotein genes (PB2, PB1, PA, and NP) from H9N2_EGY_ in the genetic background of H5N1_EGY_ was determined in vitro by minigenome assays in human 293T cells. Despite the fact that both the PB2 and the PA of H9N2_EGY_ enhance the replication of H5N1_EGY_ virus, only the PB2 polymerase subunit of H9N2_EGY_ combined with the other H5N1_EGY_ components could augment the overall in vitro polymerase activity to levels higher than those activities of the wild-type H5N1_EGY_ and H7N9_Anhui_. In the light of these results, the enhanced propagation efficiency of the H5N1_PA-H9N2EGY_ virus must be due to other mechanisms, e.g., improved functional protein interaction of the PA_H9N2EGY_ with cellular- and/or other viral proteins rather than improving the overall polymerase activity. Yet, another study showed that both PB2 and PA of a H9N2 strain (isolated from chicken in Egypt in 2013) could slightly improve the polymerase activity of another H5N1 virus (isolated from chicken in Egypt in 2013), even though not significantly [32]. As this demonstrates that the potential for H9N2 AIV genes to enhance replication of H5N1 AIV depends on specific, not-well understood mechanisms and factors, we aimed to provide a more detailed insight.

The initial site of IAV replication in the upper respiratory tract (33 °C). Normally, the internal body temperature ranges from 37 °C to 38 °C. Mild fever is occurring when body temperature ranges from 38.1–39 °C. Moderate and high grade fever is 39.1–40 °C and high-grade fever is 40.1–41.1 °C, respectively [35,36]. Drastic and irreparable changes in organ structure and function can occur when body temperature rises above 41.1 °C [36]. The temperature 39 °C was chosen to assess the viral replication of the investigated viruses as an intermediate temperature representing low-to-moderate fever during viral pneumonia. The replication efficiencies of H5N1_PB2-H9N2EGY_ and H5N1_PA-H9N2EGY_ reassortants in human cells was higher than that of the parental H5N1_EGY_ virus at 33 °C comprising the temperature of the upper respiratory tract at the initial site of infection, and at 39 °C representing the average temperature of lower respiratory tract during infection (Figure 4). This is in agreement with the work of Arai et al. [32] and strongly supports the notion that genetic recombination between H5N1- and H9N2-subtype viruses (in birds) may well yield hybrid viruses with an improved replication competence in mammalian species.

To ensure the infection of the URT and LRT of mice with the wild-type and reassortant strains, a high infectious dose “10^5^ FFU” of each virus in a relatively large volume “100 µL” was applied. We demonstrated that both reassortants H5N1_PB2-H9N2EGY_ and H5N1_PA-H9N2EGY_ did not potentiate the virulence of the wild-type H5N1_EGY_. Our data are consistent with previous studies showing that H5N1 reassortants expressing the polymerase subunits of H9N2 were less virulent in mice [32] and ferret models [33] than the respective wild-type H5N1_EGY_.

Zoonotic potential is an important characteristic that needs to be assessed for emerging animal IAV, and the reliable prediction of zoonotic potential based on nucleotide sequence data would significantly help to select for specific animal infection studies. To this point, we assessed the genetic difference between PB2_H5N1EGY_ and PB2_H9N2EGY_ and between PA _H5N1EGY_ and PA_H9N1EGY_. Two interesting variations were deduced from the sequence analyses; 591K in PB2_H9N2EGY_ and 367K in PA_H9N1EGY_ (Table 3).

The impact of these aa in H9N2, H5N1 and H7N9 strains in comparison to the fundamental 627K adaptation marker in PB2 polymerase subunit has been previously studied [28]. For this reason, we focused on the characterization of the impact of 367K in PA_H9N1EGY_ on viral replication, polymerase activity and pathogenicity of H5N1_EGY_. Notably, we found a remarkable prevalence of the lysine “K” instead of argenine “R” at aa residue 367 in human isolates of H5N1 AIV since 2014 [37]. The emergence of Q367K substitution in the field was coincident with a substantial increase in the number of human cases of H5N1 infection during the 2014–2015 flu season in Egypt [37], which may reflect its possible contribution to the overall high morbidity of avian influenza H5N1 viruses among Egyptian human population in 2014–2015. We demonstrated here for the first time that the PA_367K could improve the replication kinetics of H5N1_EGY_ in different animal cell culture models in a temperature dependent manner, especially at human URT temperature of 33 °C, regardless to the overall activity of the viral polymerase. To study the impact of the PA_367K mutation in vivo, we intentionally minimized the infectious dose of the studied viruses and the applied volume (30 µL) to ensure that the viruses were restricted to the URT of the infected mice. Interestingly PA_367K demonstrated the ability to potentiate the mortality caused by H5N1_EGY_ as demonstrated by survival rates of mice infected with mutant and wild-type viruses.

Taken together, our results demonstrate that the co-circulation of H5N1-subtype HPAIV and H9N2-subtype LPAIV in Egyptian poultry may result in co-infection and viral reassortment, thus H5N1 reassortants with an increased zoonotic potential may emerge and pose a public health risk. Specifically the lysine at position 367 of the PA seems to have a strong impact. The data presented here question the role of genetic compatibility or viral replication fitness of H5N1/H9N2 reassortants to the low or rare detection of natural genetic reassortment events among HPAI H5N1 and LPAI H9N2 viruses in Egypt. Additionally, the ability of H9N2 strains to potentiate other AIVs may be attributed to the fact that H9N2 viruses accommodate for several human adaptation markers in their genome, which may enable the reassortant AIVs to cross the species barrier.

## Figures and Tables

**Table 1:** Site directed mutagenesis primers.

Mutant Virus	Mutation	Primer Name	Mutagenesis Primer Sequence
H5N1_PA_R367K_	R367K	PA-367F	5’-GAAAAAAACGAGCCAGTTAAAGTGGGCACTCGGTGAGAACATG-3’
		PA-367R	5’-CATGTTCTCACCGAGTGCCCACTTTAACTGGCTCGTTTTTTTC-3’
H5N1_PA-H9N2EGY_K367R_	K367R	PA-K367R-H9EGY-F	5’-GAAGAAAACAAGCCAATTAAGATGGGCACTCGGTGAGAATATG-3’
		PA-K367R-H9EGY-R	5’-CATATTCTCACCGAGTGCCCATCTTAATTGGCTTGTTTTCTTC-3’

**Table 2:** Amino acid differences between PB2 and PA proteins from H5N1_EGY_ and H9N2_EGY_.

Amino Acid (aa) Residue	PB2		Amino Acid (aa) Residue	PA	
	H5N1_EGY_	H9N2_EGY_		H5N1_EGY_	H9N2_EGY_
6	E	G	38	I	V
64	I	M	58	S	G
66	I	M	94	V	I
80	R	K	101	E	D
106	A	T	129	T	I
129	N	T	184	A	V
147	T	I	204	K	R
197	R	K	212	L	R
249	K	E	269	K	R
292	M	I	287	S	A
315	I	M	321	G	N
339	T	K	323	V	A
368	Q	R	337	T	A
369	K	R	342	M	L
377	S	A	351	D	E
390	N	D	367	R	K
393	T	S	382	E	D
451	T	V	388	R	S
498	H	Q	391	K	R
521	T	A	396	D	G
529	V	I	400	T	S
570	I	M	437	H	Y
591	Q	K	448	E	A
627	K	E	450	A	V
649	I	V	539	K	R
661	T	A	554	V	I
			615	R	K
			626	R	K
			653	S	P
			669	V	I
			706	L	F
			712	A	I
			716	N	K

**Table 3:** Predominance of the distinct aa residues [28,37,38] in the studied polymerase acidic (PA) and polymerase basic 2 (PB2) proteins in Egyptian avian versus Egyptian human H5N1 isolates.

	Distinct Amino Acid (aa) Residues					
**PA Segment**	**321**	**342**	**351**	**367**	**705**	**707**
PA_H9N2EGY_	N	L	E	K	S	F
PA_H5N1EGY_	G	M	D	R	S	L
PA_H7N9Anhui_ (Human isolate)	N	L	E	K	S	F
PA_H5N1EGY_ (Human isolates 2006–2013)	S	L	E	R	S	F
PA_H5N1EGY_ (Avian isolates 2006–2013)	N	L	E	R	S	F
PA_H5N1EGY_ (Human isolates 2014–2017)	G	M	D	K	S	L
PA_H5N1EGY_ (Avian isolates 2014–2017)	N	L	E	R	Y	F
**PB2 Segment**	**591**		**627**		**701**	
PB2_H9N2EGY_	K		E		D	
PB2_H5N1EGY_	Q		K		D	
PB2_H7N9Anhui_ (Human isolate)	S		K		R	
PB2_H5N1EGY_ (Human isolates 2006–2013)	Q		K		D	
PB2_H5N1EGY_ (Avian isolates 2006–2013)	Q		K		D	
PB2_H5N1EGY_ (Human isolates 2014–2017)	Q		K		D	
PB2_H5N1EGY_ (Avian isolates 2014–2017)	Q		K		D	
